# Investigating Cavity Quantum Electrodynamics-Enabled Endo/Exo-Selectivities in a Diels–Alder Reaction

**DOI:** 10.1021/acs.jpca.5c01568

**Published:** 2025-06-13

**Authors:** Jialong Wang, Braden M. Weight, Pengfei Huo

**Affiliations:** † Department of Chemistry, 6927 University of Rochester, Rochester, New York 14627, United States; ‡ Division of Arts and Sciences, 447103 NYU Shanghai, 567 West Yangsi Road, Shanghai 200124, China; § Department of Physics and Astronomy, University of Rochester, Rochester, New York 14627, United States; ∥ The Institute of Optics, Hajim School of Engineering, University of Rochester, Rochester, New York 14627, United States; ⊥ Center for Coherence and Quantum Science, University of Rochester, Rochester, New York 14627, United States

## Abstract

Coupling molecules to a quantized radiation field inside an optical cavity has shown great promise in modifying chemical reactivity. Using the parametrized quantum electrodynamic (pQED) *ab initio* polariton chemistry approach, we theoretically demonstrate that the ground state selectivity of a Diels–Alder (DA) reaction can be fundamentally changed by strongly coupling this reaction to the cavity, generating preferential Endo or Exo isomers which are formed with equal probability for the same reaction outside the cavity. The numerical performance of pQED is in good agreement with the high-level self-consistent QED coupled cluster approach due to the exact light-matter interaction term used in pQED. By computing the ground state difference density, we show that the cavity induces a redistribution of electron density from intramolecular π-bonding orbitals to intermolecular bonding orbitals, providing chemical intuition of the cavity-induced changes to the ground state chemistry.

## Graphical abstract

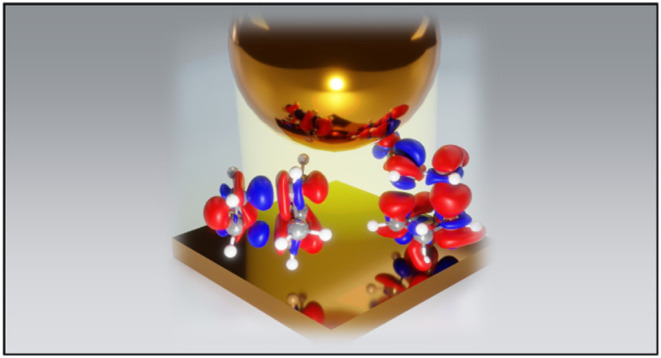

## Introduction

The Diels–Alder (DA) reaction, first elucidated in the previous century, stands as a cornerstone of organic synthesis. This cycloaddition reaction involves the formation of a conjugated diene and a dienophile, typically an alkene, culminating in a substituted cyclohexene system. DA reactions are one of the most useful techniques for creating carbon–carbon bonds.^1,2^ Furthermore, such reactions were fundamental in the Woodward–Hoffmann rules,^3^ a set of principles governing the stereochemistry of organic reactions due to the symmetry of the molecular orbitals. A common feature of DA reactions is their capacity to result in either an “Endo” or “Exo” isomer during the formation of the transition state. This results in two distinct products. More specifically, if we consider the reaction between cyclopentadiene and acrylonitrile (see Figure 1a), the resulting products under ambient conditions are known to provide Endo and Exo in equal proportion (*i.e.*, no selectivity).

**Scheme 1:**
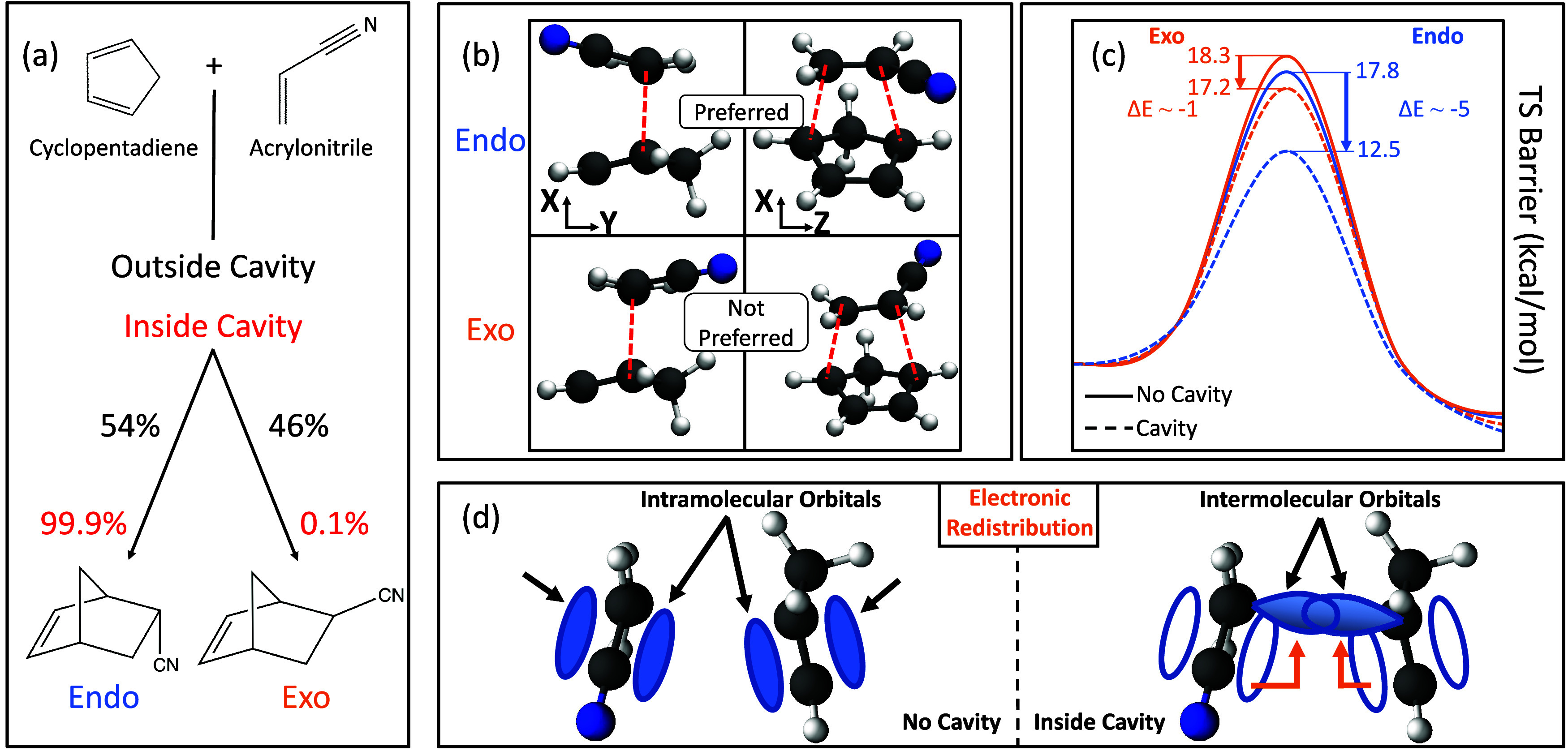
(a) Schematic Representation of the Diels–Alder Reaction between Cyclopentadiene and Acrylonitrile. The Percent Distribution of Products is Shown for the Outside (Black) and Inside (Red) of the Cavity;^4^ (b) Transition State (TS) Geometries for both Endo (Top) and Exo (Bottom) Pathways at Two Different Orientations; (c) The TS Barrier Energy Inside (Dashed) and Outside (Solid) for the Endo (Orange) and Exo (Blue) Reaction Pathways. The Cavity Polarization is Aligned with the *Y*-Direction with Light-matter Coupling Strength *A*_0_ = 0.3 a.u. and *ω*_c_ = 1.5 eV; (d) Schematic Illustration Showing the Cavity-induced Redistribution of Electron Density from Intramolecular Orbitals to Intermolecular Ones, Thus Facilitating an Intermolecular Bond and Lowering the TS Barrier Energy

It was recently proposed that strong light-matter interactions between molecules and a quantized radiation field inside an optical cavity^4^ are able to selectively produce one product over the other due to the selective change of the transition state energy. While other techniques have been proposed to selectively form the Endo or Exo products, this novel pathway opens new directions for organic and inorganic synthesis, which may pave the way for chemistry beyond what is currently accessible. However, in previous works, the molecules are placed in specific alignment with respect to the cavity polarization, and only a few calculations are performed due to the expensive QED coupled-cluster level of theoretical treatment.^4^ Here, we consider a single molecule strongly coupled to a cavity. Experimentally, this has not been achieved with Fabry–Pérot (FP) cavities. However, recent exciting progress in plasmonic cavities^5–7^ demonstrates strong coupling between the cavity and a few molecules.^5^

In this work, we use our efficient and accurate parametrized QED (pQED) approach^8,9^ to simulate how cavity QED can title the selectivities of a DA reaction. We demonstrate that the strong coupling between molecules and a cavity can fundamentally change a ground-state DA reaction. Our results suggest that one can fundamentally change the selectivity of this reaction from nonselective Endo/Exo products to highly selective Endo/Exo products by coupling this reaction inside an optical cavity. Our results obtained from pQED with linear response time-dependent density functional theory (TD-DFT) are comparable to high-level results obtained from the QED-coupled cluster level of theory.^4^ Importantly, we further provide intuitive theoretical chemical insight into the cavity-induced changes to the ground state electron density^9–12^ and relate the changes in density to the interplay between inter- and intramolecular bonding orbitals, which are commonly used in the description of bond formation.^13^

Furthermore, we compute all possible orientations of the molecule with respect to the cavity field polarization directions and identify the specific orientations of the molecule to the field polarization direction that maximizes selectivity. We find that even under isotropic disorder of the molecules with respect to the cavity polarization direction, the cavity is able to induce a significant selectivity on the order of ∼*k*_B_ *T*.

Our work demonstrates that strong coupling between molecules inside the cavity and the cavity photons offers a promising synthetic chemical tool. This coupling leads to cavity-induced changes to the ground state electron density and fundamentally modifies the outcome of known chemical reactions, making otherwise nonselective reactions selective. Our theoretical approach, pQED, offers an efficient and accurate way to simulate these reactions and provide direct chemical intuition via electron density modifications caused by coupling to the cavity.

## Theoretical Methods

We use the *ab initio* polariton approach, called parametrized-QED (pQED)^8,14^ to perform the calculations. The pQED approach uses the Pauli–Fierz (PF) Hamiltonian in the Born–Oppenheimer approximation (see eq 1) to describe light and matter interactions and use adiabatic electronic states as the basis for the electronic degrees of freedom and Fock states (*i.e.*, photon number states) as the basis for the photonic DOF. Specifically, we use the Pauli–Fierz Hamiltonian in the dipole gauge^15–18^ to investiagte how cavity vacuum fluctuations induce modifications to the ground state.^4,8–13,15,17,19–27^ The PF Hamiltonian is expressed as

1 $${Ĥ}_{\mathrm{PF}}={Ĥ}_{\mathrm{el}}+{Ĥ}_{\mathrm{ph}}+\omega_{c}A_{0}\mathrm{\mu ̂}\cdot ê\left({â}^{†}+â\right)+\omega_{c}A_{0}^{2}(\mathrm{\mu ̂}\cdot ê{)}^{2}$$

where *Ĥ*_el_ is the electronic Hamiltonian under the Born–Oppenheimer approximation (without the nuclear kinetic energy operator), *Ĥ*_ph_ = ω_c_ *â* ^†^ *â* is the Hamiltonian of the cavity field, *â* ^†^ and *â* are the raising and lowering operators of the cavity field, *ê* is a unit vector indicating the field polarization direction, and **μ̂** is the dipole operator of the molecule. The last two terms in eq 1 are the light-matter coupling (electric dipole interaction) *Ĥ*_el–ph_ = ω_c_ *A*_0_ **μ̂** · **ê**(*â* ^†^ + *â*) and the dipole-self-energy (DSE) *Ĥ*_DSE_ = ω_c_ *A*_0_^2^(**μ̂** · **ê**)^2^, respectively. Here, we assume the long-wavelength approximation such that the cavity field distribution is roughly constant across the size of the molecular reaction. We note that the existence of the DSE in the light-matter Hamiltonian has been a subject of debate in recent works with conflicting formulations of the light-matter Hamiltonian.^15,28–33^ It has been argued in ref 28, that even for pure electrostatic interactions in plasmonic cavities, the Hamiltonian should have a dipole self-energy (DSE) term which provides quadratic confinement and a bound ground state.^34^ Further, the DSE term is the consequence of performing the PZW gauge transform on the *p* · *A* Hamiltonian^15,16^ As such, we explicitly include the DSE term in this work, as was done in the previous work of scQED-CCSD in ref 4.

Moreover, The light-matter coupling strength is expressed as

2 $$A_{0}=\sqrt{\frac{1}{2\omega_{c}\epsilon V}}$$

where ϵ is the permittivity inside the cavity, and V is the effective mode volume. Alternatively, the electric field strength ε = ω_c_ *A*_0_ can be used as a measure of coupling strength, which is common in experiments. In state-of-the-art cavity designs, such as those from gold or silver nanoparticle-on-metal (NPoM) cavities, the local electric field can vary from 1 to 10 V/nm,^6,7^ which is well within the cavity parameters used in the present work. We chose ω_c_ = 1.5 eV and the coupling strength *A*_0_ = 0.3 au that is equivalent to a mode volume V∼ 0.19 nm^3^ and field intensity of E∼ 8.50 V/nm, which the cavity frequency and field strength are experimentally achievable for plasmonic nanocavity parameters.^6,7^ We emphasize that for plasmonic cavities, in addition to the transverse fields, there are direct Coulomb interactions between the atoms of the plasmonic nanoparticles (or surface) and the molecule in question.^29^ The degree to which the electrostatic interactions or the coupling to the transverse field is important and will depend on the distance between the molecules and the metal surface (or nanoparticle) surface and on how fast the evanescent field decays. We emphasize that the direct Coulomb interactions between the atoms of the plasmonic nanoparticles and the molecule are not included in the current theoretical model. Here, we make this choice to be consistent with the previous theoretical work using scQED-CCSD^4^ such that we can directly benchmark our pQED-TDDFT results with those obtained using high-level scQED-CCSD theory (see Figure 2) at the same level of Hamiltonian. Future work is needed to investigate the influence of the direct Coulomb interactions, likely using the framework of Macroscopic QED^29,35^ or by directly including the plasmonic particle in the electronic/polaritonic structure calculation.^36,37^

As we discussed in our previous work,^8,9,17^ the following two couplings in the Hamiltonian^15–17^ shown in eq 1 cause the polariton ground states modifications. First, the off-resonance light-matter term (*Ĥ*_el–ph_) couples through the ground state permanent dipole and transition dipoles between the ground and excited states. One simple example for the first case is the coupling between |ψ_g_, 0⟩ and |ψ_g_, 1⟩, which is propotional to ⟨ψ_g_, 0|μ̂(*â* ^†^ + *â*)|ψ_g_, 1⟩=μ_gg_⟨0|(*â* ^†^ + *â*)|1⟩ = μ_gg_, and |ψ_g_, 1⟩ will further couple to |ψ_e_, 0⟩ through terms like ⟨ψ_e_, 0|μ̂(*â* ^†^ + *â*)|ψ_g_, 1⟩ = μ_ge_⟨0|(*â* ^†^ + *â*)|1⟩, where μ_gg_ and μ_ge_ are the permenant and transition dipoles among the ground and excited states, each projected along the cavity polarization direction **ê**. The usual notion of hybrid light-matter states arise from this coupling term when the molecular ground state with one photon |ψ_g_, 1⟩ and the excited molecular state with zero photons |ψ_e_, 0⟩ become close in energy and hybridize into |Φ_e_⟩ ∝ |ψ_g_, 1⟩ + |ψ_e_, 0⟩.^8,15^

The second contribution is from the DSE, which does not couple states of varying photon numbers but does provide nontrivial electronic couplings between electronic ground and excited states. The DSE terms that couples to the ground state are proportional to ⟨ψ_g_|μ̂^2^|ψ_α_⟩ = ∑_γ_μ_*g*γ_μ_γ*α*_, where α and γ include the ground and all excited electronic states. Overall, the direct coupling and DSE terms, *Ĥ*_el–ph_ and *Ĥ*_DSE_, both contribute to modifications to the ground state.^8,9,17,21,28,34,38,39^ Through these nonresonant light-matter couplings, the cavity induces modifications to the reactions that are beyond the prediction of the simple Jaynes–Cummings model.^40^

The polariton eigenstates and eigenenergies are obtained by solving the following eigenvalue equation

3 $${Ĥ}_{\mathrm{PF}}\left|\Phi_{j}\left(R\right)\right\rangle =E_{j}\left(R\right)\left|\Phi_{j}\left(R\right)\right\rangle$$

where *Ĥ*_PF_ is given in eq 1, *E*_*j*_(**R**) are the Born–Oppenheimer polaritonic potential energy surfaces (PES) (which parametrically depend on the nuclear coordinates **R**), and |*E*_*j*_(**R**)⟩ are the adiabatic polariton states. We directly diagonalize the polaritonic Hamiltonian *Ĥ*_PF_ matrix and obtain the eigenvalues. The basis is constructed using the tensor product of electronic adiabatic states |ψ_α_(**R**)⟩(*i.e.*, eigenstates of the electronic Hamiltonian ${Ĥ}_{\mathrm{el}}\left|\psi_{\alpha }\left(R\right)\right\rangle =E_{\alpha }\left(R\right)\left|\psi_{\alpha }\left(R\right)\right\rangle$ ) and the Fock states |*n*⟩ (*i.e.*, eigenstates of the photonic Hamiltonian *Ĥ*_ph_|*n*⟩ = *nω*_c_|*n*⟩), expressed as |ψ_α_(**R**)⟩ ⊗ |*n*⟩ ≡ |ψ_α_(**R**), *n*⟩. This basis is used to evaluate the matrix elements of *Ĥ*_PF_, and diagonalizing it provides *E*_*j*_(**R**) and the corresponding polariton states

4 $$\left|\Phi_{j}\left(R\right)\right\rangle =\sum_{\alpha }^{N_{\mathrm{el}}}\sum_{n}^{N_{F}}C_{\alpha n}^{j}\left|\psi_{\alpha }\left(R\right),n\right\rangle$$

where *C*_α*n*_^*j*^ = ⟨ψ_α_ (**R**), *n*|Φ_*j*_ (**R**)⟩. Here, the number of included electronic states, $N_{\mathrm{el}}$ , and photonic Fock/number states, $N_{F}$ , are treated as convergence parameters.

In the Diels–Alder reaction investigated in this work, the numbers of states we used to solve the eq 3 are $N_{F}=10$ and $N_{\mathrm{el}}=50$ . We use the light-matter coupling strength *A*_0_ = 0.3 au and coupling frequency ω_c_ = 1.5 eV to perform the reaction. We have carefully checked the convergence of the calculation following the procedure outlined in our previous works.^8,9^ Further details regarding the pQED approach and higher coupling frequency results are provided in the Supporting Information.

All electronic structure computations were performed using the Q-CHEM software package.^41^ We employed the parametrized quantum electrodynamics time-dependent density functional theory (pQED-TDDFT) approach with the ωB97XD hybrid exchange-correlation functional and the 6–311+G** basis set. When aligning the cavity polarization direction **ê** with a specific molecular axis, either **ê** = **X** or **ê** = **Y**, or **ê** = **Z**, the matrix elements ⟨ψ_α_|**μ̂** · **X**|ψ_γ_⟩ and ⟨ψ_α_|**μ̂** · **Y**|ψ_γ_⟩ are input for the interaction term **μ̂** · **ê** and for the DSE term. For the cavity polarization direction in a general case (see Figure 4), the interaction term follows the relationship **ê** · **μ̂** = sin θ cos ϕ **X** · **μ̂** + sin θ sin ϕ **Y** · **μ̂** + cos θ **Z** · **μ̂**. Both ground state energies and electron density differences were determined using the Q-CHEM package.^41^

## Results and Discussions

We investigate the DA reaction between cyclopentadiene and acrylonitrile (Scheme 1a). This reaction produces two distinct Endo/Exo isomers as products. Outside the cavity and under standard reaction conditions, the DA reaction is kinetically controlled and shows a nonselective result with 54% Endo to 46% Exo products. It has been recently proposed^4^ that this intrinsically nonselective reaction can be made selective by coupling the ground state of the reacting molecules to an optical cavity with frequency in the range of electronic excitations (*i.e.*, ω_c_ ∼ 1–3 eV) in contrast to the recently explored vibrational strong coupling regime^42,43^ (*i.e.*, ω_c_ ∼ 0.1 eV).

Scheme 1 highlights the main results of this work, with the reaction depicted in panel (a). Panel (b) shows the transition states (TS) of this reaction that lead to the Endo (top) or the Exo (bottom) products. The red dashed lines between the molecules show the bonds that will form upon the reaction. Furthermore, we emphasize that the Endo pathway becomes preferred inside the cavity under experimentally feasible cavity conditions, even in the presence of orientational disorder of the molecule with respect to the cavity field polarization direction. As suggested in ref 4. (and confirmed in the current work), the selectivity shifts to 99.9% for the Endo product and only 0.1% for the Exo. As an example of the modifications to the PES, we show the ground state PES in Scheme 1c, where the reactant (R) and TS geometries of the Endo (blue) and Exo (orange) isomers are placed inside the cavity with the cavity polarization along the *Y*-direction of the molecule. In this case, there is a significant change of the selectivity toward Endo species through a reduction of the TS barrier height by ∼5 kcal/mol for the Endo and ∼1 kcal/mol for the Exo compared to outside the cavity. This shifts the expected yields of the reaction to 99.9 and 0.1% for the Endo and Exo isomers, respectively, consistent with previous work in ref 4. In this case, our pQED-TDDFT calculations quantitatively reproduced the scQED-coupled cluster with singles and doubles excitations (QED-CCSD) approach,^4^ with more details and comparisons to be discussed in Figure 2. This shift in selectivity can be understood as cavity-induced electronic redistribution under the influence of the cavity (Scheme 1d). More specifically, coupling to the cavity induces electron density to be taken from occupied intramolecular π-bonding orbitals (*i.e.*, single-particle orbitals) to virtual intermolecular orbitals, thus facilitating a reduction in energy of the TS barrier height.

Figure 1 presents the *X*-, *Y*-, and *Z*-directions of cavity field polarization using the TS geometries provided in ref 4. Figure 1 shows the TS barrier height *E* ^‡^ = *E*_0_(**R**_TS_) – *E*_0_(**R**_reac_) in the ground polaritonic state |Φ_0_(**R**)⟩ as a function of the light-matter coupling strength *A*_0_ for the three primary cavity field polarization directions, (blue) *X*, (orange) *Y*, and (green) *Z*, for the (a) Endo and (b) Exo isomers. In both the Endo and Exo pathways (Figure 1), the TS barrier increases for the *X*-polarized cavity (blue curve) by 7.2 and 6.7 kcal/mol at *A*_0_ = 0.3 au, respectively, compared to outside the cavity (*A*_0_ = 0.0 au). The X-polarized cavity is not expected to offer selectivity for this reaction due to the simultaneous and unfavorable increase in TS barrier energy for the two isomers. In contrast, the *Y*-direction shows a decrease in both the Endo (5.3 kcal/mol) and the Exo (1.3 kcal/mol) pathways. The Endo isomer exhibits an additional 3.0 kcal/mol reduction in the TS barrier compared to the Exo isomer, thus offering a significant selectivity toward the Endo isomer. The *Z*-direction also offers a cavity-mediated selectivity, now favoring the Exo isomer. In this case, the Endo isomer’s TS barrier is increased by 1.5 kcal/mol, while the Exo barrier height is decreased by 1.8 kcal/mol, generating a 3.3 kcal/mol difference in TS barrier height between isomers. In the Y- and Z-polarization cases, we expect the Endo product yields to be $P_{\mathrm{Endo}}=\exp\left[-E_{\mathrm{Endo}}^{‡}/k_{B}T\right]/Z$ = 99.9 and 0.4%, respectively, where $Z=\exp\left[-E_{\mathrm{Endo}}^{‡}/k_{B}T\right]+\exp\left[-E_{\mathrm{Exo}}^{‡}/k_{B}T\right]$ . Thus, the theoretical results demonstrate that the cavity can offer a novel approach toward the selective isomerization of this DA reaction.

**Figure 1:**
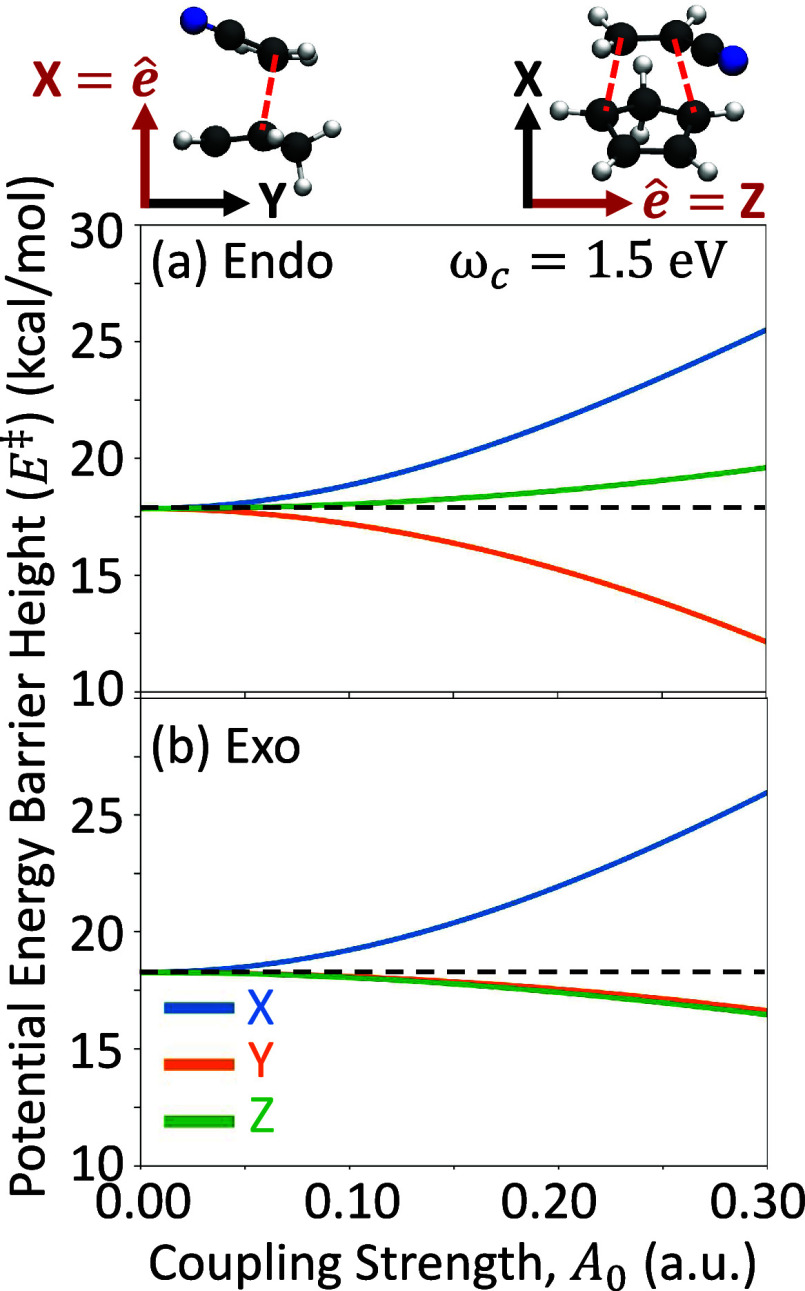
Polaritonic ground state activation energy, defined as the energy difference between the transition state and the reactant geometries, *E* ^‡^ = *E*_0_(**R**_TS_) – *E*_0_(**R**_reac_), for the two reaction pathways, (a) Endo and (b) Exo. Here, *E*_0_(**R**) is the polaritonic ground state energy defined in eq 3 at nuclear geometry **R**. The colors correspond to cavity polarizations along the *X*- (blue), *Y*- (orange), and *Z*-directions (green). The cavity frequency is ω_c_ = 1.5 eV. The horizontal dashed line indicates the uncoupled barrier height (*i.e.*, *A*_0_ = 0.0 au).

Figure 2 presents a direct comparison between the pQED-TDDFT of the current work using pQED-TDDFT and that of the high-level scQED-CCSD of ref 4. Here, the solid lines represent results obtained from scQED-CCSD, and the dashed lines represent the results from our pQED-TDDFT approach. Note that there are only three data points for each curve, reporting relative energies for reactant (R), transition state (TS), and product (P), and the curves are interpolations (with an interpolated spline grid portraying the rest of the potential energy surface) that provide visual guidance. Black curves represent the case outside the cavity, and the red curves represent the case inside the cavity. Overall, our pQED results agree semiquantitatively with the accurate and expensive scQED-CCSD in terms of predicting the relative trend of barrier modifications for inside and outside the cavity cases. In general, we find only minor quantitative differences between the two approaches that can be rationalized by the known deviations between standard CCSD and DFT methodologies, which are expected to reach 1–5 kcal/mol. Here, such deviations reach up to 3.0 kcal/mol for the Endo pathway and 2.6 kcal/mol for the Exo pathway, signifying that our pQED-TDDFT is well within the expected error of the bare many-body approach itself.^8^ More importantly, our pQED-TDDFT results portray the same semiquantitative behavior of the Endo and Exo potential energy surfaces as the scQED-CCSD for all data points except two: the *X*- and *Z*-polarization directions for the Endo product energies. In the *X*-polarization direction, the scQED-CCSD approach predicts an increase in energy for the Endo product, while our pQED-TDDFT method indicates a slight decrease. In the *Z*-polarization direction, the scQED-CCSD results show a minor decrease in product energy, whereas the pQED-TDDFT approach shows an increase. A more detailed analysis of these subtle differences is available in the Supporting Information. Furthermore, the differences in the QED-CCSD and pQED-TDDFT energies are less than 2 kcal/mol and well within the error expected between the standard TDDFT and CCSD methodologies and thus acceptable for our qualitative exploration of this DA reaction which, for the rest of the work, only focuses on the correctly reproduced TS barrier geometries/energies.

**Figure 2:**
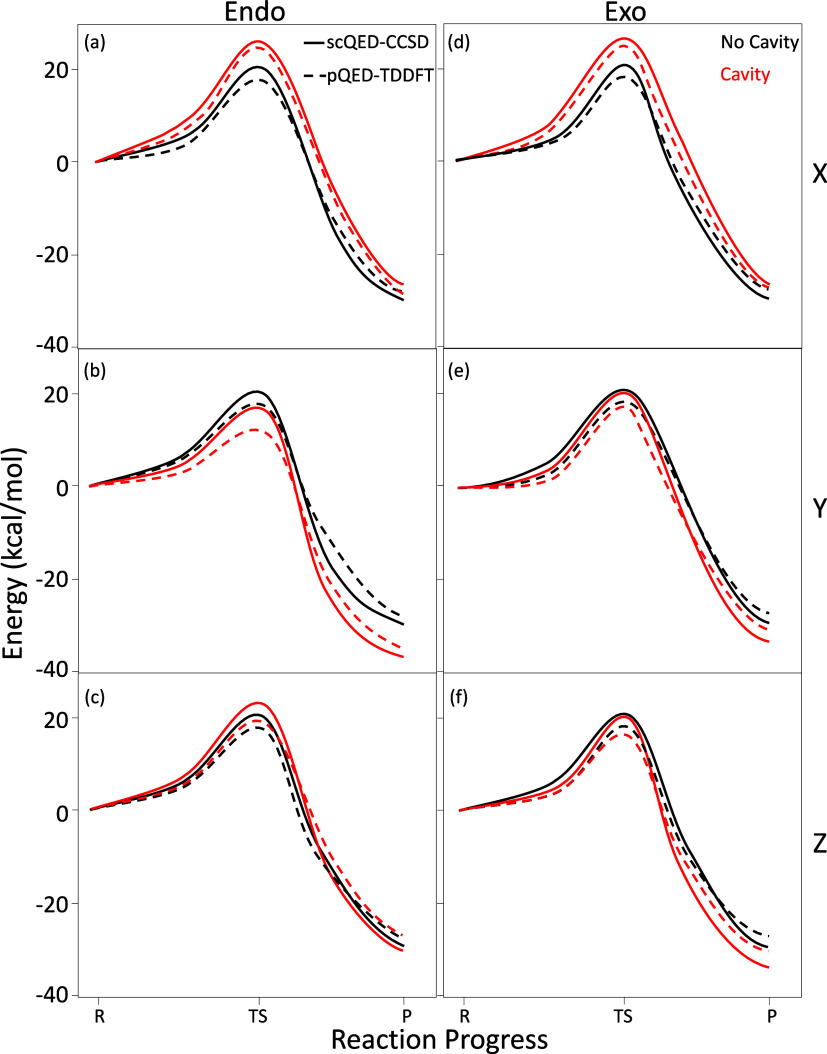
Potential energy surfaces, *E*_0_(**R**), as functions of the reaction progress from the reactant (R) to the transition state (TS) and to the product (P), inside (red) and outside (black) the cavity for both reaction pathways (a–c) Endo and (d–f) Exo. The (a, d) *X*, (b, e) *Y*, and (c, f) *Z* polarizations of the cavity are shown. The (dashed) pQED-TDDFT approach of the current work is directly compared to the (solid) scQED-CCSD method of ref 4. The curves were interpolated between the R, TS, and P data points using a spline approach to improve visual clarity. The light-matter coupling strength is *A*_0_ = 0.3 au with cavity frequency ω_c_ = 1.5 eV.

To rationalize the observations seen in Figures 1 and 2 (which was not presented in the earlier work of ref 4). Figure 3 shows the density difference isosurfaces^9,10^ for the TS geometries for the Endo (top) and Exo (bottom) isomers for all three principle cavity polariation directions: *X* (left), *Y* (middle), and *Z* (right). The difference density function is defined as Δ*ρ*_00_(**r**) = ρ_00_^M^(**r**) – ξ_00_(**r**), where ρ_00_^M^ = Tr_ph_[ρ̂_00_] = Tr_ph_[|Φ_0_⟩⟨Φ_0_|] is the total ground state polaritonic density with the photon DOFs traced out. ξ_00_(**r**) = ψ_0_^*^(**r**) ψ_0_(**r**) is the bare electronic ground state density. The difference between these two densities portrays the effects of cavity-induced electronic redistribution around the molecule. The regions in which Δ*ρ*_00_(**r**) > 0 (red colored) indicate that a gain of electron density has occurred and depletion when Δ*ρ*_00_(**r**) < 0 (blue colored). Additional visualization angles are shown in Figure S4 in the Supporting Information. This effect can be rationalized via chemical intuition by considering that the cavity can induce redistribution (exchange of character) between bare occupied and unoccupied single-particle orbitals (*e.g.*, HOMO ↔ LUMO), which allows for changes to the standard molecular orbital theory inside the cavity.^13^

**Figure 3:**
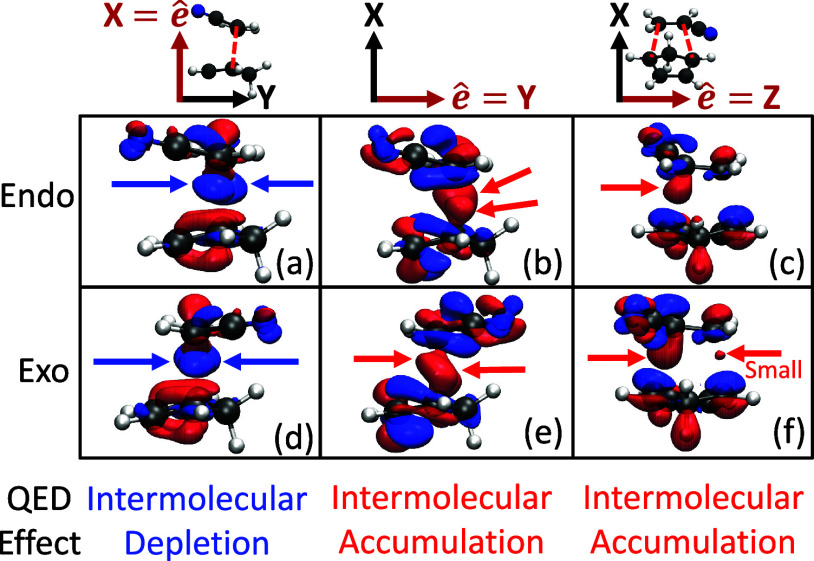
Difference density isosurfaces of the transition state geometries for the (a–c) Endo and (d–f) Exo pathways. The cavity polarization direction is along the (a, d) *X*-, (b, e) *Y*-, and (c, f) *Z*-directions. The isosurfaces in each panel correspond to the difference density, Δ*ρ*_00_(*x*, *y*, *z*) = ρ_00_^M^(*x, y, z*) – ξ_00_(*x, y, z*), at the transition state geometry using the pQED-TDDFT approach of the current work. The color indicates the accumulation (red) or depletion (blue) of electron density upon coupling to the cavity. The arrows indicate the corresponding change of electron density that can be interpreted as “intermolecular bonding orbitals”. In all cases, the light-matter coupling strength *A*_0_ = 0.2 au with cavity frequency ω_c_ = 1.5 eV. The isovalue chosen for the *X*-direction is 1.0 and 0.2 *m*|*e*|/Å^2^ for the *Y*- and *Z*-polariations, where *m*|*e*| = |*e*| × 1000 and |*e*| is the charge of an electron.

The *X*-polarization direction showcased a simultaneous increase in TS barrier energy for the Endo and Exo isomers (see Figure 1), thus, we expect that the potential chemical bond between the two reactant molecules is weakened by the presence of the cavity for both isomer configurations. Figure 3a,d show the ground state difference density isosurface for the Endo (Figure 3a) and Exo (Figure 3d) isomers with the cavity polarized along the *X*-direction of the molecule (see Cartesian axes above Figure 3a). The region between the reactant molecules is blue, which indicates that this region has been depleted of electron density. This region is also responsible for the formation of the intermolecular bond during the reaction. Since this region has lost these intermolecular bonding electrons, the TS geometry has been destabilized compared to outside the cavity. Contrary to this result, the *Y*-polarization of the cavity induced a stabilization of the TS barrier energy (Figure 1). Figure 3b,e show the difference density in this case, and, opposite to Figure 3a,d, we find an increase in electron density in the region between the reactant species, this strengthening the intermolecular bond at the TS geometry and reducing the TS barrier energy.

The regions not localized between the reactant species in Figure 3 are considered as intramolecular density redistributions. These density differences have a similar shape as intramolecular π-bonding orbitals. This is especially evident in the cyclopentadiene molecule. For the *X*-polarization, these orbitals exhibit electron density accumulation from the intermolecular bonding orbitals. For the *Y*-polarization, on the other hand, these intramolecular π-bonding orbitals donate their electrons to the intermolecular bond. Thus, the effects of the cavity are to induce changes to the bonding structure of the reactant species, thus either enhancing or weakening the bond formation depending on the cavity polarization direction.

The *Z*-polarization direction is weakly changing the TS barrier energy (Figure 1) and oppositely between the Endo and Exo isomers. Notably, the difference density in this case (Figure 3c,f) exhibits weaker and asymmetric changes to the intermolecular region. Note that the molecule is rotated by 90 deg about the *X*-axis in Figure 3c,f compared to Figure 3a,b,d,e for visual clarity. Additionally, the intramolecular density, especially on the bottom molecule of the figure (cyclopentadiene) shows a different symmetry compared to those shown in Figure 3a,b,d,e where the underside of the intramolecular π-bonds are accumulating electron density while the top side is being depleted. Overall, the redistribution of electron density does not facilitate the formation of the two covalent bonds and thus showcases a weaker change to the TS barrier height compared to the *X*- and *Y*-polarization directions.

Overall, we have used the difference density function to develop a chemically appealing interpretation of the cavity-modified DA reaction between cyclopentadiene and acrylonitrile. In particular, the cavity-mediated redistribution of charges closely resembles the inter- and intramolecular bonding orbitals. The electron density is explicitly modified by the cavity to facilitate the intermolecular bonds by donating electron density from intramolecular π-orbitals (largely localized on the cyclopentadiene species) to the forming intermolecular bond. The intermolecular bonds can instead be weakened by the interactions with the cavity by removing electron density from the intermolecular bonds and donating it to the intramolecular bonds.

The cavity polarization directions along the principal Cartesian axes (*X*, *Y*, and *Z*) were taken as a benchmark from the previous work of ref 4. However, the use of these Cartesian directions as “important” field polarization directions is a theoretical choice and may be difficult to control in experiments, despite the exciting progress on using supermolecular host–guest chemistry when coupling a single molecule with the plasmonic cavity.^5^ With this in mind, we explore an arbitrary cavity field polarization vector *ê* = *ê*(ϕ, θ), where ϕ and θ are the azimuthal and polar angles, respectively, defined schematically in Figure 4a. Figure 4b,c show the change in TS barrier energy $\Delta E^{‡}\left(\phi ,\theta \right)=E^{‡}\left(\phi ,\theta \right)-E^{‡}$ for the Endo and Exo isomer, respectively, with light-matter coupling strength *A*_0_ = 0.3 au and cavity frequency ω_c_ = 1.5 eV. Here, *E* ^‡^(ϕ, θ) is the polaritonic ground state TS barrier energy and $E_{0}$ is the bare electronic ground state TS barrier energy (equivalent to *E* ^‡^ with *A*_0_ = 0.0 au and ω_c_ = 0.0 eV). The negative regions indicate a reduction in the TS barrier height inside the cavity, while the red regions show an increase in the TS barrier height.

**Figure 4:**
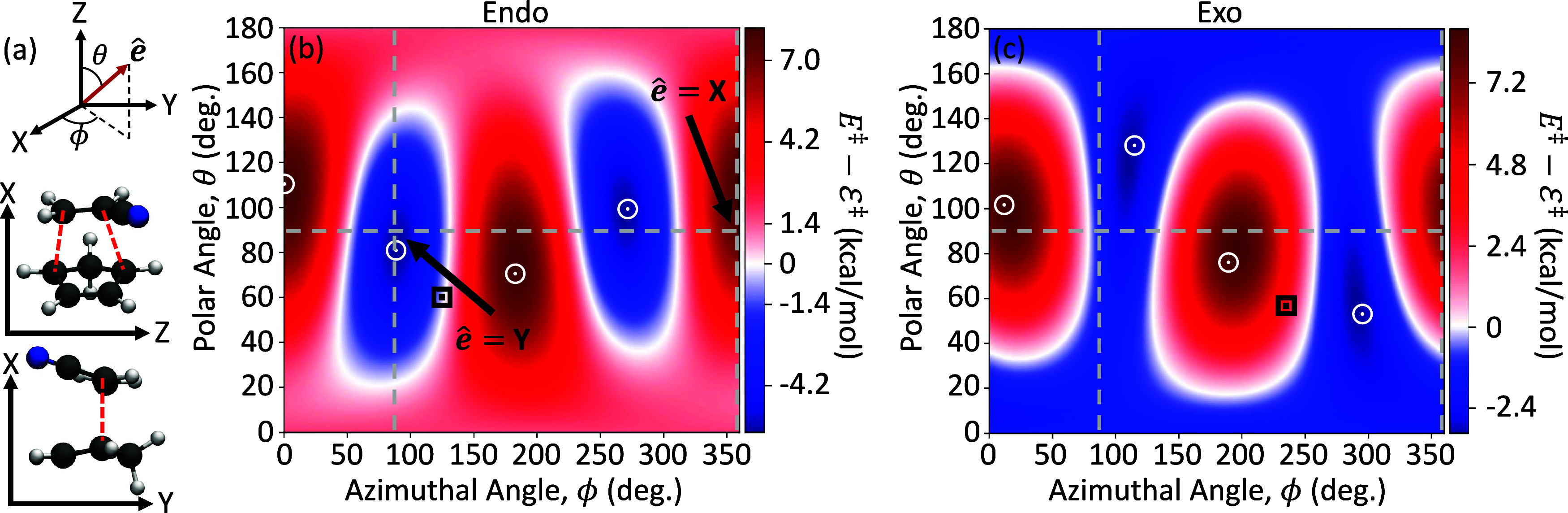
(a) Schematic illustration of the spherical coordinate system with an arbitrary cavity polarization vector *ê*(ϕ, θ) and two orientations of the molecule with respect to the primary Cartesian axes. (b, c) The difference between the polaritonic transition state barrier, *E* ^‡^ = *E*_0_(**R**_TS_) – *E*_0_(**R**_reac_), and the barrier of the bare molecular system, $E^{‡}=E_{0}\left(R_{\mathrm{TS}}\right)-E_{0}\left(R_{\mathrm{reac}}\right)$ , for the (b) Endo and (c) Exo reaction pathways as functions of the azimuthal ϕ and polar θ angles. The color bar indicates the sign and magnitude of the difference of the energy barrier height, $E^{‡}-E^{‡}$ . The blue regions indicate where the transition state barrier is lowered compared to outside the cavity. The white symbols indicate the maxima and minima values, with only two nondegenerate points on each pathway, and are related to the other set by symmetry. For both panels, the light-matter coupling strength *A*_0_ = 0.3 au and cavity frequency ω_c_ = 1.5 eV.

In Figure 4b, the Endo isomer at certain values of (ϕ, θ) has a TS barrier energy that is maximized (white circle in the red region) and minimized (white circle in the blue region) for this choice of cavity parameters. We define these special configuration points as (ϕ_1_, θ_1_) = (3.3°, 111.2°) and (ϕ_2_, θ_2_) = (91.7°, 80.2°), respectively. Connecting to the previous figures, the *X*- and *Y*-directions are equivalent to (ϕ, θ) = (0°, 90°) = (360°, 90°) and (ϕ, θ) = (90°, 90°) = (270°, 90°), respectively. In the Endo case (Figure 4b), the *X*- and *Y*-polarization directions are near to the critical points (ϕ_1_, θ_1_) and (ϕ_2_, θ_2_). However, for the Exo isomer (Figure 4c), the critical points are located at (ϕ_3_, θ_3_) = (11.5°, 103.1°) and (ϕ_4_, θ_4_) = (114.6°, 126.1°). Hence, the *Y*-axis direction is far from either of the extrema for the Exo case. In fact, the *Y*-direction lies on the border between the stabilizing region (blue) and the destabilizing region (red). In both cases, the *Z*-direction is far from any critical point, implying that this direction of cavity polarization is not optimal in either isomer. Later, in Figure 7, the *Z*-polarization is shown to still be valuable in cavity-induced selectivity even though both isomers, individually, experience a mediocre cavity effect. We found the maximum and minimum critical points for the Endo pathway to be (ϕ_1_, θ_1_) = (ϕ_MAX_^Endo^, θ_MAX_^Endo^) = (3.3°, 111.2°) and (ϕ_2_, θ_2_) = (ϕ_MIN_^Endo^, θ_MIN_^Endo^) = (91.7°, 80.2°); for Exo pathway, the points are (ϕ_3_, θ_3_) = (ϕ_MAX_^Exo^, θ_MAX_^Exo^) = (11.5°, 103.1°) and (ϕ_4_, θ_4_) = (ϕ_MIN_^Exo^, θ_MIN_^Exo^) = (114.6°, 126.1°), respectively. The black square symbols indicate the ground state dipole moment unit vectors, μ⃗_00_, for the Endo and Exo pathways, which are (Figure 4b) (125.6°, 60.8°) and (Figure 4c) (235.7°, 56.9°), respectively.

Figure 5 shows the TS barrier energy *E* ^‡^ as a function of the light-matter coupling strength *A*_0_ for the above-mentioned critical angles for the cavity polarization vector (ϕ_*i*_, θ_*i*_) for both isomers. The cavity frequency is ω_c_ = 1.5 eV. It is evident that the (ϕ_1_, θ_1_) and (ϕ_3_, θ_3_) maximize the individual isomer TS barrier energies while the (ϕ_2_, θ_2_) and (ϕ_4_, θ_4_) minimize this energy for all values of coupling strength *A*_0_. In turn, we can inspect the ground state difference density isosurfaces for these critical points, as shown in Figure 6. As expected, the polarization angles that maximize the TS barrier energy contain intermolecular electron density depletion, destabilizing the forming bond, as well as electron accumulation in the intramolecular bonding π-orbitals of each reactant molecule. The opposite is again true for the angles that minimize the TS barrier energy, showing electron density accumulation in the intermolecular bonding region. Notably, the intramolecular π-bonding orbitals showcase asymmetric accumulation/depletion, similar to the *Z*-polarization in Figure 3c,f. We hypothesize that these critical angles of the field induce a complicated redistribution of electron density, not only from the reactant species to the forming “intermolecular bond” but also among themselves in a way that further decreases the energy of the TS geometry. Hence, examining only the principle directions *X*, *Y*, and *Z* as defined by chemical intuition will mostly likely not showcase the maximal effects of the complicated electron-photon correlation (as the black square symbols shown in Figure 4) since the direction of the many coupled permanent and transition dipole matrix elements in the adiabatic electronic basis is not straightforward and likely does not relate to a simple and meaningful chemical property.

**Figure 5:**
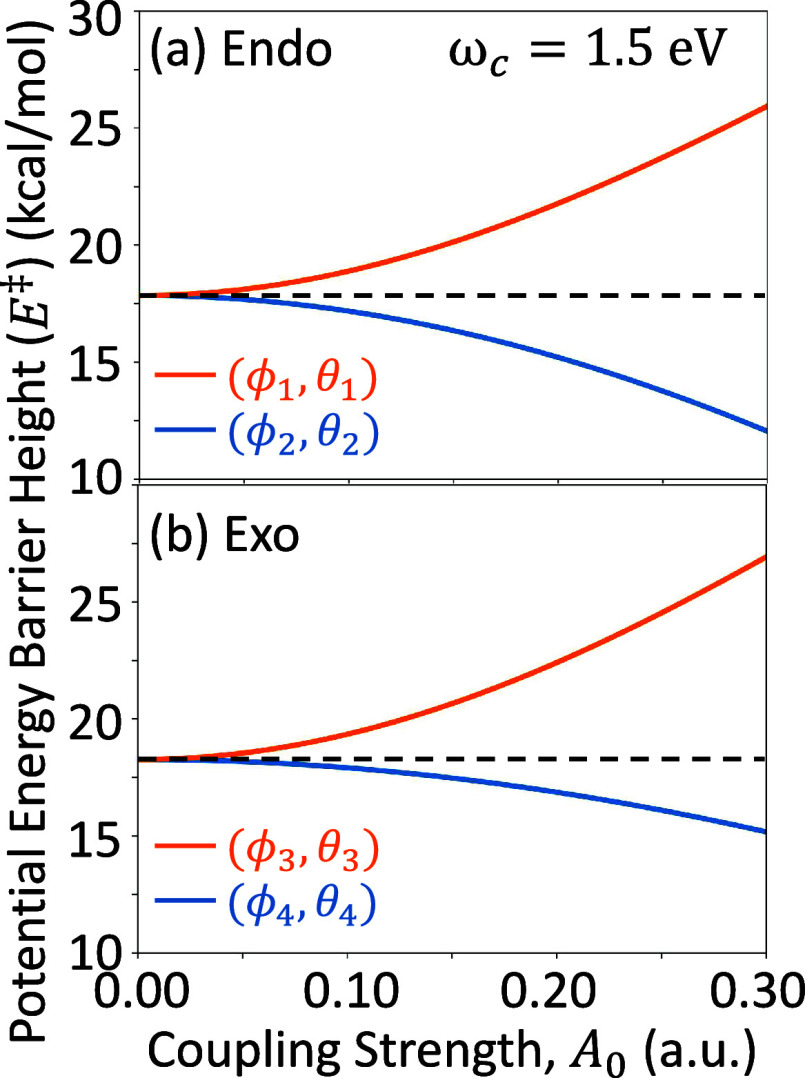
Polaritonic ground state activation energy, defined as the energy difference between the transition state and the reactant geometries, *E* ^‡^ = *E*_0_(**R**_TS_) – *E*_0_(**R**_reac_), for the two reaction pathways, (a) Endo and (b) Exo. The cavity polarizations are shown at the critical points for each pathway: (ϕ_1_, θ_1_) and (ϕ_3_, θ_3_) (MAX in orange); (ϕ_2_, θ_2_) and (ϕ_4_, θ_4_) (MIN in blue). The cavity frequency is ω_c_ = 1.5 eV. The horizontal dashed line indicates the uncoupled barrier height (*i.e.*, *A*_0_ = 0.0 au).

**Figure 6:**
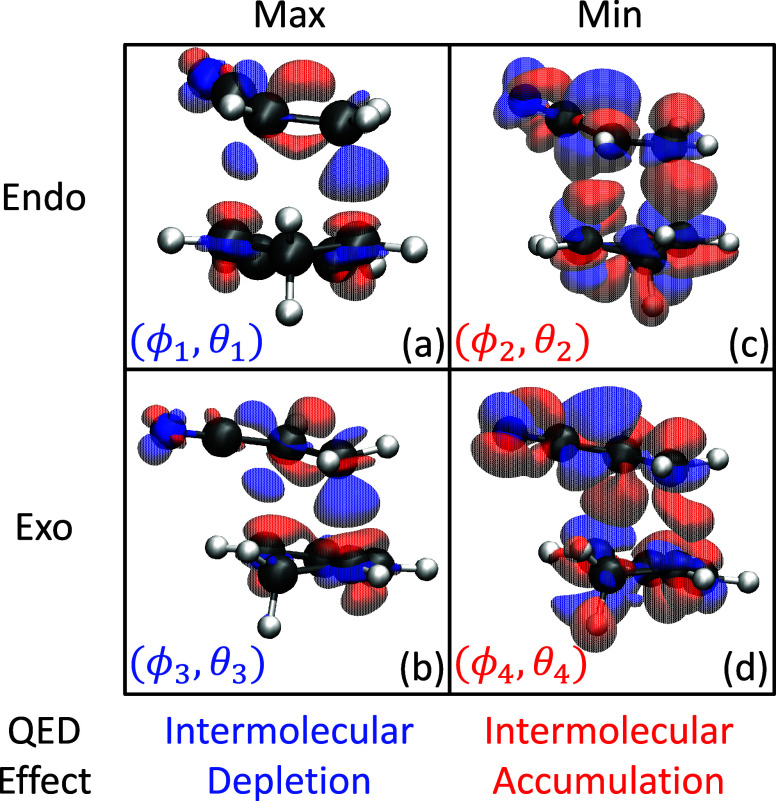
Difference density isosurfaces at the transition state geometries for the (top) Endo and (bottom) Exo pathways. The cavity polarizations are shown at the critical points for each pathway: (a) MAX, Endo pathway (ϕ_1_, θ_1_); (b) MAX, Exo pathway (ϕ_3_, θ_3_); (c) MIN, Endo pathway (ϕ_2_, θ_2_); (d) MIN, Exo pathway (ϕ_4_, θ_4_). The color indicates the accumulation (red) or depletion (blue) of electron density upon insertion into the cavity. The arrows indicate the intermolecular bonding orbitals. In all cases, the light-matter coupling strength *A*_0_ = 0.2 au with cavity frequency ω_c_ = 1.5 eV. The isovalue chosen for both maxima is 1.0 and 0.2 *m*|*e*|/Å^2^ for the minima, where *m*|*e*| = |*e*| × 1000 and |*e*| is the charge of an electron.

Figure 7a presents the TS barrier energy difference, *E*_Endo_^‡^ – *E*_Exo_^‡^, as a function of the cavity polarization direction (ϕ, θ) for a fixed cavity frequency ω_c_ = 1.5 eV and light-matter coupling strength *A*_0_ = 0.3 au This figure depicts the energy difference between the barrier heights of the two isomers, *E*_Endo_^‡^ – *E*_Exo_^‡^. Thus, Figure 7 is related to the probability of forming either Endo or Exo species at a given orientation of the molecule with respect to the cavity field direction. Negative values of this quantity (blue regions) indicate parameter regimes where the Endo pathway is lower in TS energy compared to the Exo pathway. Contrary to this, positive values indicate regions where the Exo pathway TS has a lower energy. The cavity polarization angles at which the highest amount of selectivity toward the Endo, (ϕ_Endo_, θ_Endo_) = (68.8°, 80.2°) at 5.88 *k*_B_ *T*, and Exo, (ϕ_Exo_, θ_Exo_) = (137.5°, 160.4°) at −10.73 *k*_B_ *T* are the critical points. These angles are shown as white circle-dots in Figure 7a.

**Figure 7:**
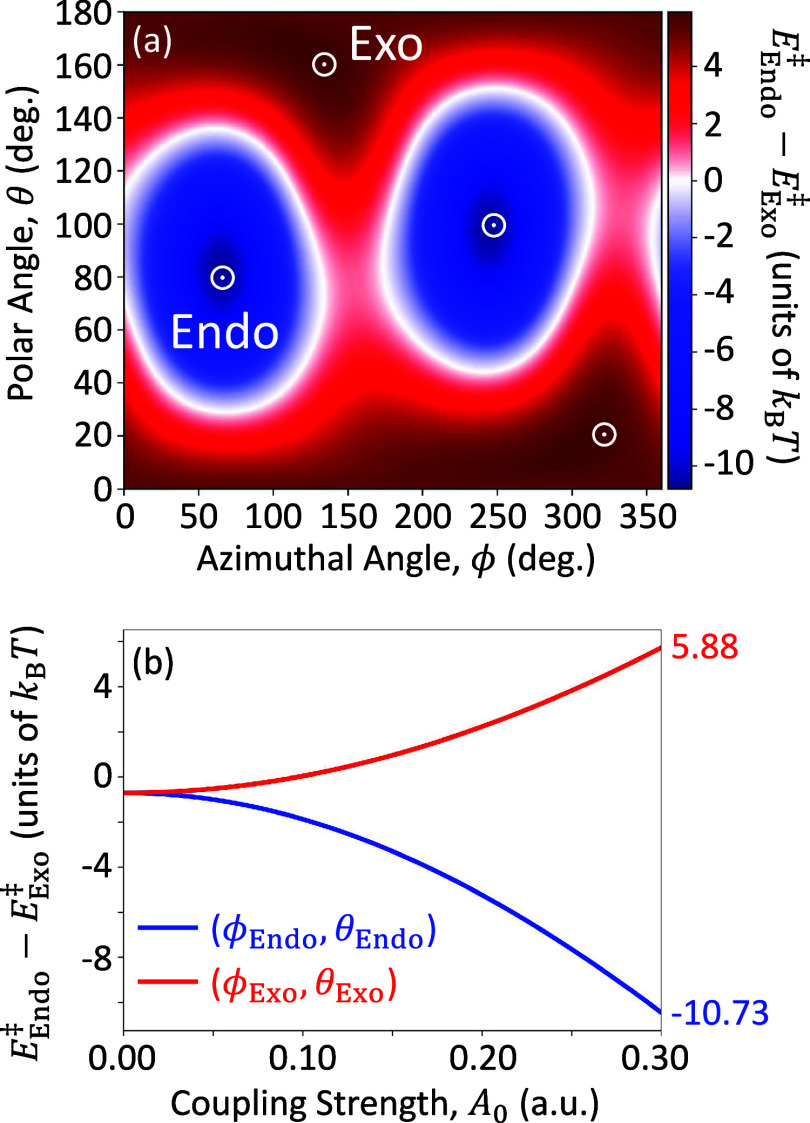
(a) TS energy difference between the Endo and Exo isomers, *E*_Endo_^‡^ – *E*_Exo_^‡^, as a function of the cavity polarization direction, (ϕ, θ). The blue region (*E*_Endo_^‡^ < *E*_Exo_^‡^) indicates that the Endo pathway has a lower barrier energy and is preferable compared to the Exo pathway; the red region, in contrast, indicates that the Exo pathway is preferable. The light-matter coupling strength *A*_0_ = 0.3 au and the cavity frequency ω_c_ = 1.5 eV. The white circle-dot symbols indicate the critical points at which *E*_Endo_^‡^ – *E*_Exo_^‡^ is maximized, (ϕ_Exo_, θ_Exo_) = (137.5°, 160.4°), or minimized, (ϕ_Endo_, θ_Endo_) = (68.8°, 80.2°), offering the maximum amount of selectiveity for the Exo and Endo isomers, respectively. (b) TS barrier energy difference between the Endo and Exo isomers, *E*_Endo_^‡^ – *E*_Exo_^‡^ as a function of the light-matter coupling strength at the critical angles which produce the maximal selectivity of Endo (blue) and Exo (red) isomers.

In experiments, control over the light-matter coupling strength *A*_0_ is difficult and is often susceptible to many environmental factors. While our calculations predict strong selectivity at these critical angles of cavity polarization direction, the selectivity at weaker light-matter coupling strengths *A*_0_ may provide a deeper insight into experimental observations. Figure 7b presents the TS barrier energy difference, *E*_Endo_^‡^ – *E*_Exo_^‡^, as a function of the light-matter coupling strength *A*_0_ for both of the critical angles shown in Figure 7a. At small values of light-matter coupling (*A*_0_ < 0.05 au), negligible selectivity change is predicted. Our calculations predict that, at these critical angles, prominent Endo selectivity can be achieved at or above *A*_0_ = 0.10 au at which the TS barrier energy difference is greater than 2 *k*_B_ *T* at room temperature. For the Exo isomer, the selectivity is weaker and requires at least *A*_0_ = 0.20 au for the same degree of selectivity induced by the TS barrier energy difference. Hence, in the experiment, strong selectivity in the reaction is already achievable with current plasmonic cavity designs.^6,7^

Furthermore, while experimentally feasible,^5^ it is often difficult to control the orientation of the molecules with respect to the cavity’s electric field polarization (ϕ, θ). In the experiment, we expect a random orientation of the molecules (isotropic disorder). We calculate the angular average of a cavity modified observable *O*(ϕ, θ) as follows

5 $$\left\langle O\left(\phi ,\theta \right)\right\rangle =\frac{\int \sin\theta d\theta \int d\phi O\left(\phi ,\theta \right)}{\int \sin\theta d\theta \int d\phi }$$

For example, the average transition state energy difference between the Endo and Exo isomers is ⟨*E*_Endo_^‡^ – *E*_Exo_^‡^⟩ = −0.9212 *k*_B_ *T* at room temperature (300 K) using data in Figure 7a. This implies that, even by considering the isotropic disorder, the Endo pathway is still preferred by nearly one *k*_B_ *T* at room temperature, whereas for outside the cavity case, there should be an equal mixture of the Endo and Exo products. Hence, we have theoretically shown that this DA reaction will provide appreciable selectivity inside the cavity, even if the orientation of the molecules cannot be controlled.

Finally, we investigate the individual contributions to the cavity-induced selectivity of this DA reaction. Figure 8 presents the contributions from individual terms in eq 1 to the TS energies at the critical cavity polarization angles for the Endo (Figure 8a,c) and Exo (Figure 8b,d) isomers. The energy contributions are calculated as *E*_*a*_^‡^ = ⟨Φ_0_(**R**_TS_)|*Ĥ*_*a*_|Φ_0_(**R**_TS_)⟩ – ⟨Φ_0_ (**R**_reac_)|*Ĥ*_*a*_|Φ_0_(**R**_reac_)⟩, where *Ĥ*_a_ ∈ {*Ĥ*_PF_, *Ĥ*_el_, *Ĥ*_ph_, *Ĥ*_el–ph_, *Ĥ*_DSE_}. Further, |Φ_0_(**R**)⟩ is the ground state polaritonic wave function, always defined by the total PF Hamiltonian *Ĥ*_PF_|Φ_0_(**R**)⟩= *E*_0_(**R**) |Φ_0_(**R**)⟩(eq 3). These contributions are shown for each of the four cavity polarization directions defined in Figure 4: (ϕ_1_, θ_1_) in Figure 8a, (ϕ_3_, θ_3_) in Figure 8b, (ϕ_2_, θ_2_) in Figure 8c, and (ϕ_4_, θ_4_) in Figure 8d. These angles represent the largest increase (Figure 8a,b) and largest decrease (Figure 8c,d) in the transition state energy for the Endo (Figure 8a,c) and Exo (Figure 8b,d) configurations. The cavity frequency is set to be ω_c_ = 1.5 eV. In the Supporting Information, Figures S5 and S6 present the same data but for the reactant and TS geometries, individually.

**Figure 8:**
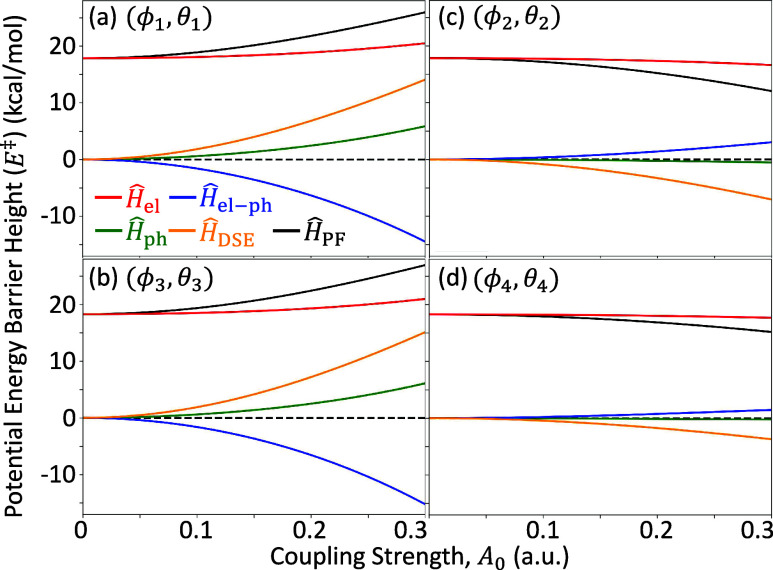
Energy contributions from individual terms from *Ĥ*_PF_ in eq 1 to the ground state energy barrier height for each of the four extrema points (a–d) defined as white circles in Figure 4. Components are *Ĥ*_el_ in red, *Ĥ*_el–ph_ in blue, *Ĥ*_ph_ in green and *Ĥ*_DSE_ in gold. The horizontal dashed black line indicates the barrier height changes outside the cavity (*i.e.*, *A*_0_ = 0.0 au). The cavity frequency is ω_c_ = 1.5 eV.

By construction, the total energy *Ĥ*_PF_ (solid black curve) for (ϕ_1_, θ_1_) and (ϕ_3_, θ_3_) increases as a function of the light-matter coupling strength and decreases for (ϕ_2_, θ_2_) and (ϕ_4_, θ_4_). Of most importance and interest are the two interaction terms *Ĥ*_el–ph_ (solid red curve) and *Ĥ*_DSE_ (solid gold curve), which are responsible for the modifications to the TS barrier energy *E* ^‡^ inside the cavity. For both critical angles at which the TS barrier energy is maximized (Figure 8a,b), the DSE contributes positively to the energy while the direct electron-photon interaction provides a negative contribution. Note that the energy of the DSE for a single molecule coupled to a cavity is a positive contribution, while the bilinear interaction term is contributes negatively to the ground state energy. Here, we are showing the energy difference between two nuclear geometries, *E* ^‡^ = *E*_TS_ – *E*_reac_, for which the contribution of either term can be positive or negative (see Figures S5 and S6 in the Supporting Information for the absolute energies of each term).

For the critical angles in which the TS barrier energy is minimized (Figure 8c,d), the opposite trends are observed, where the DSE contributes negatively while the direct interaction term is positive. Additionally, the magnitudes of all terms are reduced since the cavity-induces TS barrier decreases (negative values/blue in Figure 4) are less in magnitude than the cavity-induced increases (positive values/red in Figure 4). From Figure 8, it is clear that the DSE is directly related to the chemically relevant modifications to the ground state energies, since the DSE contribution nearly quantitatively reproduces the changes to the TS barrier energy in all cases (*i.e.*, other contributions largely cancel among each other). This also provides confirmation of the various mean-field QED-HF calculations^10,13,23,24,26,27,44^ as well as high-level approaches^4,10–12,19,20,22,25,45–49^ in the community exploring ground state cavity-modifications.^8,9,17,21,28,34,38,39^ Furthermore, we expect that these results are largely independent of any strong resonance effects. Thus, modulating the cavity frequency ω_c_ will only influence the light-matter coupling as a scaling factor. This was discussed in our previous work.^9,17^

## Conclusions

We theoretically investigated the cavity modification on a textbook ground state Diels–Alder (DA) reaction. By coupling to a quantized cavity radiation field, one can selectively generate one type of the product (Endo or Exo) compared to the outside the cavity case (under standard reaction conditions) where the reaction produces an equal mixture of both products. Our results demonstrate that the cavity induces significant selectivity toward the Endo isomer, even for moderate coupling strength, as well as for random molecular orientations (isotropic disorder). In addition, we have shown that the pQED-TDDFT method semiquantitatively agrees with the high-level scQED-CCSD approach^4^ and with errors between the two approaches less than 3 kcal/mol.

By computing the ground state difference density, we show that the cavity induces a redistribution of electron density to stabilize or destabilize the TS geometry, depending on the cavity polarization direction. Cavity-induced stabilization occurs by shifting electron density from intramolecular π-bonding orbitals to intermolecular bonding orbitals. Destabilization occurs through the opposite mechanism, where the intermolecular bonding orbitals donate their electron density to intramolecular π-bonding orbitals. Our results have provided chemically relevant insights into the cavity-induced changes to the ground state chemistry and, thus, changes to the molecular orbital theory inside the cavity.^13^ While the specific chemically intuitive reason for the discrimination of the Endo and Exo isomers is still unclear, we believe the answer to be in the details of the squared dipole matrix μ̂^2^, specifically the differences in this matrix between the Endo and Exo isomers at the transition state geometry. The exploration and discussion of this is beyond the scope of the current work and will be the topic of a future work by us.

We further explore an arbitrary molecular orientation relative to the cavity polarization direction, which leads to critical polarization angles that maximize the Endo or Exoselectivity of the reaction. Here, we show that the optimal selectivity for the ground state reaction, in terms of the cavity polarization direction, does not correspond to a simple chemically relevant direction but involves a complicated interplay between the many permanent and transition dipole orientations of the reacting molecules. Overall, we show that maximum selectivity for the Endo and Exo isomers can be achieved with relative barrier energies approaching ∼5 and ∼10 *k*_B_ *T*, respectively. Even when assuming isotropic disorder in the orientation of the molecule with respect to the cavity polarization direction, we find that the Endo isomer is still preferred by ∼*k*_B_ *T*, which is significantly different than the situation outside the cavity where both products are equally probable.

Finally, we decompose the individual energy contributions from the PF Hamiltonian (in eq 1) and provide a discussion on the effects of the dipole self-energy on the polaritonic ground state. The DSE contribution to the TS barrier energy has identical trends with the energy of the total Hamiltonian. Thus, we conclude that the DSE is the leading order physics to the cavity-mediated ground state modifications in this particular DA reaction, which is in agreement with many other works at the mean-field QED-HF level and beyond.^4^ We hope this work enables further study of ground state chemistry inside the cavity that includes (i) identification of the optimal cavity polarization direction for each reaction (ii) a quantitative benchmark against other approaches, and (iii) a detailed comparison of the cavity parameters with state-of-the-art experimental cavity designs.

## Supplementary Material



## Notes

Light-matter coupling strength scans, pQED-TDDFT/scQED-CCSD benchmark comparisons, three-dimensional isosurfaces of the difference density, and energy contributions of reactant and transition state geometries (PDF)
