# Supplementary material for: Investigating Cavity Quantum Electrodynamics-Enabled Endo/Exo-Selectivities in a Diels–Alder Reaction
Source: J Phys Chem A. 2025 Jun 13;129(25):5458–68. doi: 10.1021/acs.jpca.5c01568 (PMC12207675; doi:10.1021/acs.jpca.5c01568)
Supplement: Supplementary file 1 [file jp5c01568_si_001.pdf]

Supporting Information: Investigating Cavity Quantum Electrodynamics-Enabled Endo/Exo- Selectivities in a Diels-Alder Reaction

Jialong Wang,[†,‡] Braden M. Weight,[*,¶] and Pengfei Huo[*,†,§,‖]

†*Department of Chemistry, University of Rochester, Rochester, NY 14627, U.S.A.*

‡*Division of Arts and Sciences, NYU Shanghai, 567 West Yangsi Road, Shanghai 200124, China*

¶*Department of Physics and Astronomy, University of Rochester, Rochester, NY 14627, U.S.A.*

§*The Institute of Optics, Hajim School of Engineering, University of Rochester, Rochester, NY 14627, U.S.A.*

‖*Center for Coherence and Quantum Science, University of Rochester, Rochester, New York 14627, U.S.A.*

E-mail: bweight@ur.rochester.edu; pengfei.huo@rochester.edu

We explored the X-, Y-, and Z-directions of cavity field polarization using the TS geometries provided in Ref. 4 at $\omega_c = 10.0$ eV. Using higher coupling frequency, a stronger TS barrier increases or decreases are observed, as shown in Fig. S1. For X-polarized cavity (blue curve), the TS barrier increases by 16.1 kcal/mol and 17.9 kcal/mol; compared to Fig. 1 in main text that $\omega_c = 1.5$ eV brings a 7.2 kcal/mol and 6.7 kcal/mol, we observe a more than a 120% increase because of the increase of coupling frequency. However, the X-polarized cavity is still not expected to offer selectivity for this reaction due to the simultaneous and unfavorable increase in TS barrier energy for these two isomers. Same trends for Y- and Z-polarized cavity are observed when comparing this stronger ω_c to the lower, experimental achievable one in the main text. The disparity in TS barrier height for the Z-direction between the Endo and Exo pathways — 5.4 kcal/mol increase for the endo isomer, and 5.1 kcal/mol decrease for the exo isomer is still observed.

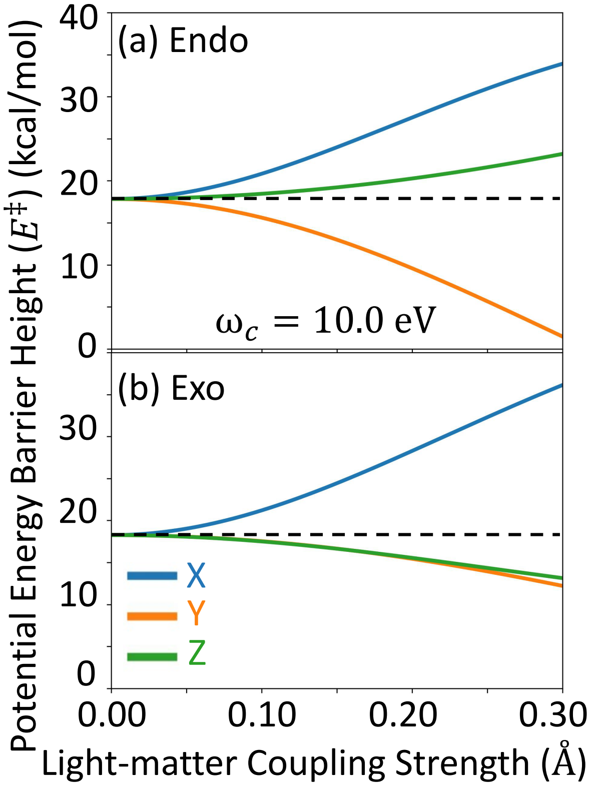

Figure S1: The polaritonic ground state activation energy, defined as the energy difference between the transition state and the reactant geometries, $E^{\ddagger} = E_0(\mathbf{R}_{\mathrm{TS}}) - E_0(\mathbf{R}_{\mathrm{reac}})$, for the two reaction pathways, (a) Endo and (b) Exo. Here, $E_0(\mathbf{R})$ is the polaritonic ground state energy defined in Eq. 1 in the main text at nuclear geometry $\mathbf{R}$. The colors correspond to cavity polarizations along the X- (blue), Y- (orange) and Z-directions (green). The cavity frequency is $\omega_{\mathrm{c}} = 10.0$ eV. The horizontal dashed line indicates the uncoupled barrier height (*i.e.*, $A_0 = 0.0$ a.u.).

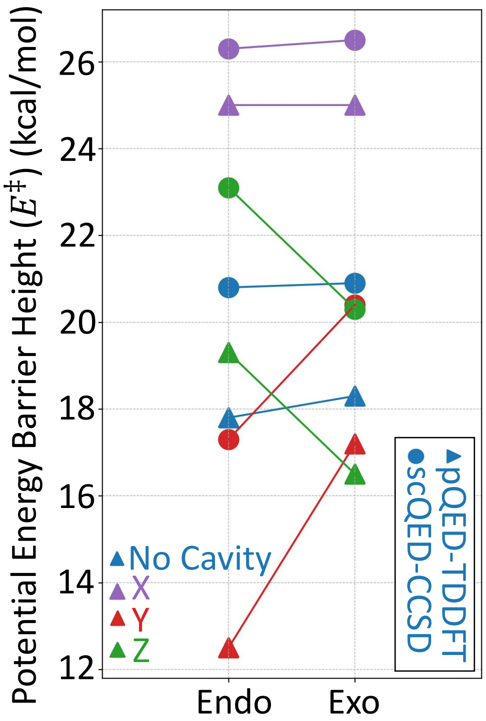

Figure S2: TS barrier height comparison between pQED-TDDFT and scQED-CCSD methods. The color indicates different polarization, with X- (violet), Y- (red), Z-directions (green) and outside cavity (blue). The pQED-TDDFT (triangle) approach of the current work is directly compared to the scQED-CCSD method (dot) of Ref. 4. The light-matter coupling strength is $A_0 = 0.3$ a.u. with cavity frequency $\omega_c = 1.5$ eV.

Through Fig. S2, we can clearly see how TS barrier for both endo (left) and exo isomers (right). Compare both reactions without cavity (blue), we can find for both Endo and Exo, pQED-TDDFT (blue traingle) is close to scQED-CCSD method (blue dot) from Ref. 4. For the X-polarization (violet), the two methods have even less error bar; a more clear comparision between the difference of these two methods is shown in Fig. S3, which the discussion in next part. Checking the Y-directions in red, we can observe for our method (in triangle) gives a 12.5 kcal/mol for endo isomer and 17.2 kcal/mol for exo isomer, compare to the scQED-CCSD method which shows a 17.3 kcal/mol and 20.4 kcal/mol, respectively. This already gives us a clear picture that pQED-TDDFT is nearly close to the scQED-CCSD.

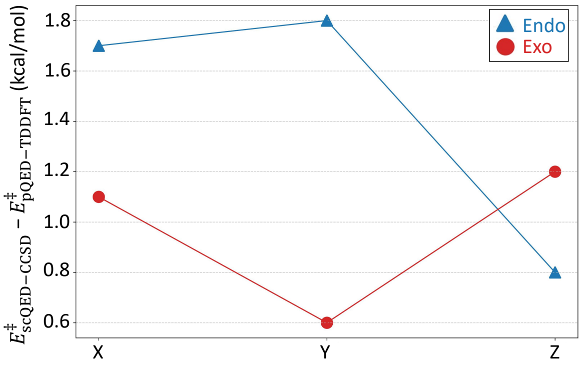

Figure S3: TS barrier height difference between pQED-TDDFT and scQED-CCSD methods. The symbol indicates differnt isomers, endo (blue triangle) and exo (red dot). The light-matter coupling strength is $A_0 = 0.3$ a.u. with cavity frequency $\omega_c = 1.5$ eV.

Moreover, in Fig. S3, for example, in the X-direction, the shifts are also within 1.3 and 1.5 kcal/mol for the Endo and Exo pathways, respectively, using both methods. Given that the differences reported in our work fall within this range while reproducing the same qualitative trends for the TS barrier change, we believe this work benchmarking successfully with the methods mentioned in the Ref. 4. Overall, we observe that TS barrier has lower shifts for exo isomers than endo isomers when comparing these two methods, especially for the Y-direction, the shift for Exo pathway falls less than 1 kcal/mol. Therefore, we believe that our TDDFT method is convincible with the high-level scQED-CCSD method.

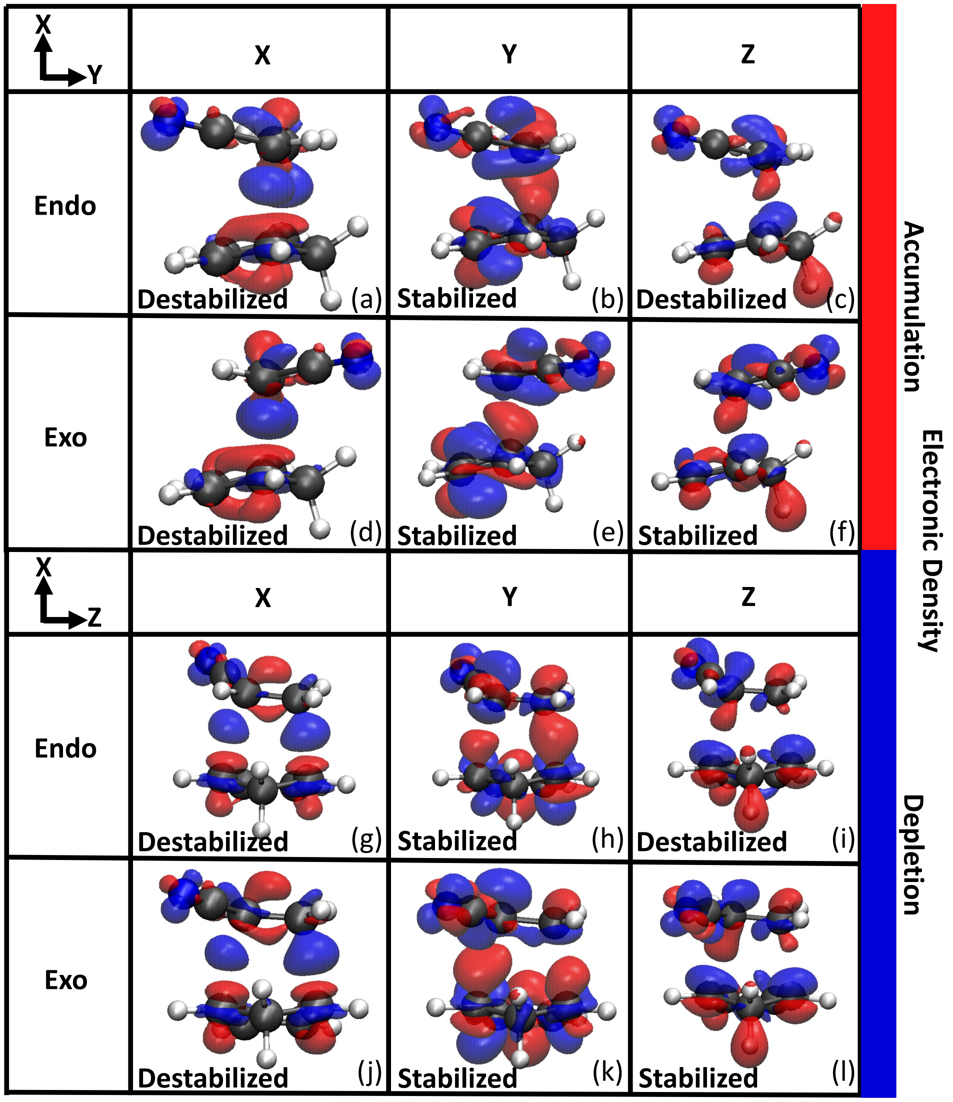

Figure S4: Difference density isosurfaces at the transition state geometries for endo and exo isomers, viewing as (a-f) X-Y perspective and (g-l) X-Z perspective for cavity polarizations along the (a, d, g, j) X-, (b, e, h, k) Y-, and (c, f, i, l) Z-directions. The isosurfaces in each panel correspond to the difference density, $\Delta\rho_{00}(x,y,z) = \rho^{\mathrm{M}}_{00}(x,y,z) - \xi_{00}(x,y,z)$, at the transition state geometry using the pQED-TDDFT approach of the current work. The color indicates the accumulation (red) or depletion (blue) of electron density upon insertion into the cavity. In all cases, the light-matter coupling strength $A_0 = 0.2$ a.u. with cavity frequency $\omega_{\mathrm{c}} = 1.5$ eV. The isovalue chosen for the X-direction is 1.0 m|e|/$\mathring{A}^2$ and 0.2 m|e|/$\mathring{A}^2$ for the Y- and Z-polariations, where m|e| = |e|×1000 and |e| is the charge of an electron.

We visualize difference density isosurfaces in Figure. 3 (main text) in differnt angles, as there are two different perspectives in Fig. S4. In the X-directions at X-Z perspective, we can observe a more clear picture of the recharge of electron distrbution for both (Fig. S4g)

endo and (Fig. S4j) exo isomers. The deleption of electron desnity, which is the blue region is clearly shown in between the two reacants at the TS geomertries. Thus, the lose of these intermolecular bonding electrons makes TS geometry destabilized compared to outside the cavity. Moreover, in Fig. S4 (h) and (i), we can find a better angle that shows the increase in electron desnity in the reaction region, indicating the increase of intermolecular bond strength and thus decrease in the TS barrier height. As discussed in the main text, the empty region between the two reactants are due to the intramolecular density redistributions. We can conclude that the effects of the cavity facilitate the bond the formation or disallow it through the redistrbution of electrons.

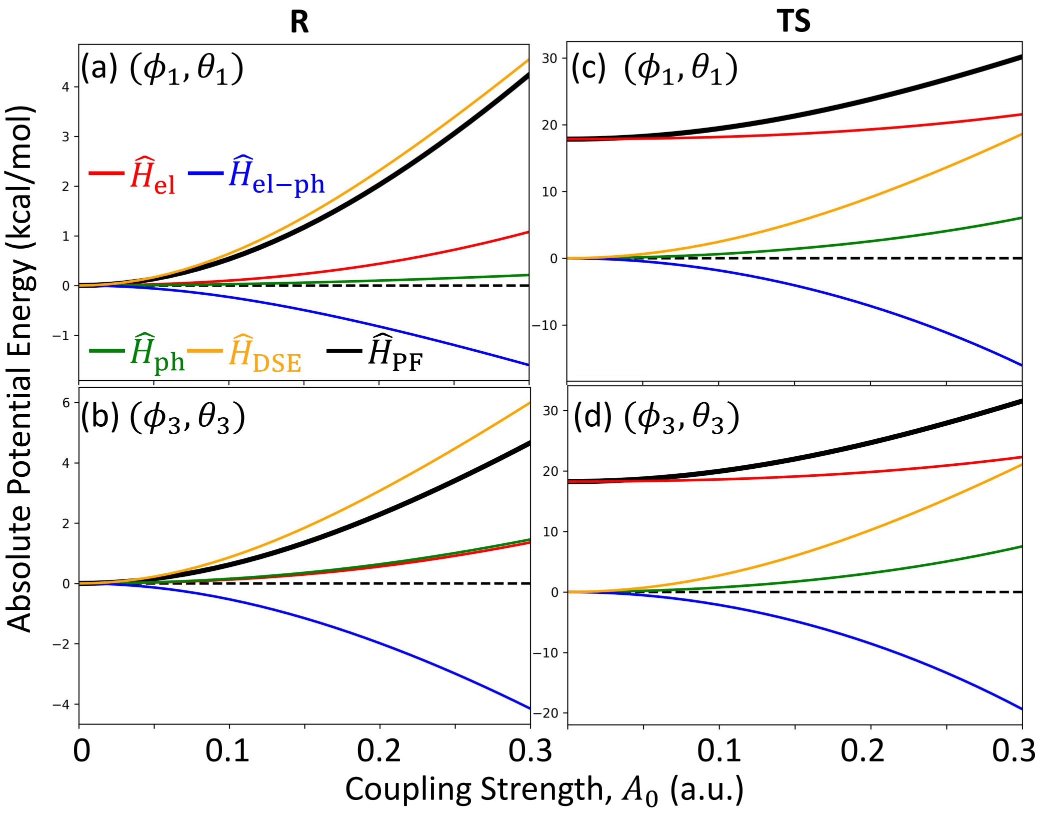

Figure S5: Absolute barrier energy contribution for R and TS to the ground state energy barrier height at maxmimal difference of $E^{\ddagger} - \mathcal{E}^{\ddagger}$. (a, c) (ϕ_1, θ_1), (b, d) (ϕ_3, θ_3). (a) and (b) show aboslute energy for R; (c) and (d) show aboslute energy for TS. It shows different energy contirbutions towards the total Hamiltonian $\hat{H}_{\text{PF}}$, which is in black solid curved line. Components are $\hat{H}_{\text{el}}$ in red, $\hat{H}_{\text{el-ph}}$ in blue, $\hat{H}_{\text{ph}}$ in green and $\hat{H}_{\text{DSE}}$ in yellow. The horizontal dashed line indicates the uncoupled barrier height (*i.e.*, $A_0 = 0.0$ a.u.). Note that $\hat{H}_{\text{el}}$ starts at the same barrier height as $\hat{H}_{\text{PF}}$ since we treated the molecular Hamitonian at that zero-point level. Overall, $\hat{H}_{\text{DSE}}$ contributes the most to the total energy. The cavity frequency is $\omega_{\text{c}} = 1.5$ eV.

Fig. S5 and Fig. S6 show the abosulte energy contributions of each Hamiltonian componets for R and TS geomerties, seperately. Both figures present the absolute R (a,b in Fig. S6 and Fig. S6) and TS barrier energies (c,d in Fig. S6 and Fig. S6) at the critical cavity polarization angles for the endo (Fig. S5a,c and Fig. S6a,c) and exo (Fig. S5b,d and Fig. S6b,d) isomers.

The energy contributions mentioned in the main text are shown for each of the four cavity polarization directions defined in: (Fig. S5a,c) (ϕ_1, θ_1), (Fig. S5b,d) (ϕ_3, θ_3), (Fig. S6a,c) (ϕ_2, θ_2), and (Fig. S5b,d) (ϕ_4, θ_4). In other words, these angles represent the largest (Fig. S5) increase and (Fig. S6) decrease in the energy. Since these are the same components before

making the TS barrier height in Fig. 8 in the main text, all the individual components follow the same trend.

The energy contributions from $\hat{H}_{\mathrm{el}}$ (solid red curve) and $\hat{H}_{\mathrm{ph}}$ (solid green curve) terms are activated indirectly by the coupling terms and are more difficult to attribute to simple physics, except that as the absolute energy of $\hat{H}_{\mathrm{el}}$ and $\hat{H}_{\mathrm{ph}}$ increases in a given nuclear configuration, increasing the contribution from higher-lying electronic excited states and higher photon number states to the polaritonic ground state. In all four cases of critical angles, the cavity-induced changes to these terms is smaller than the ther direct electron-photon interaction and DSE terms.

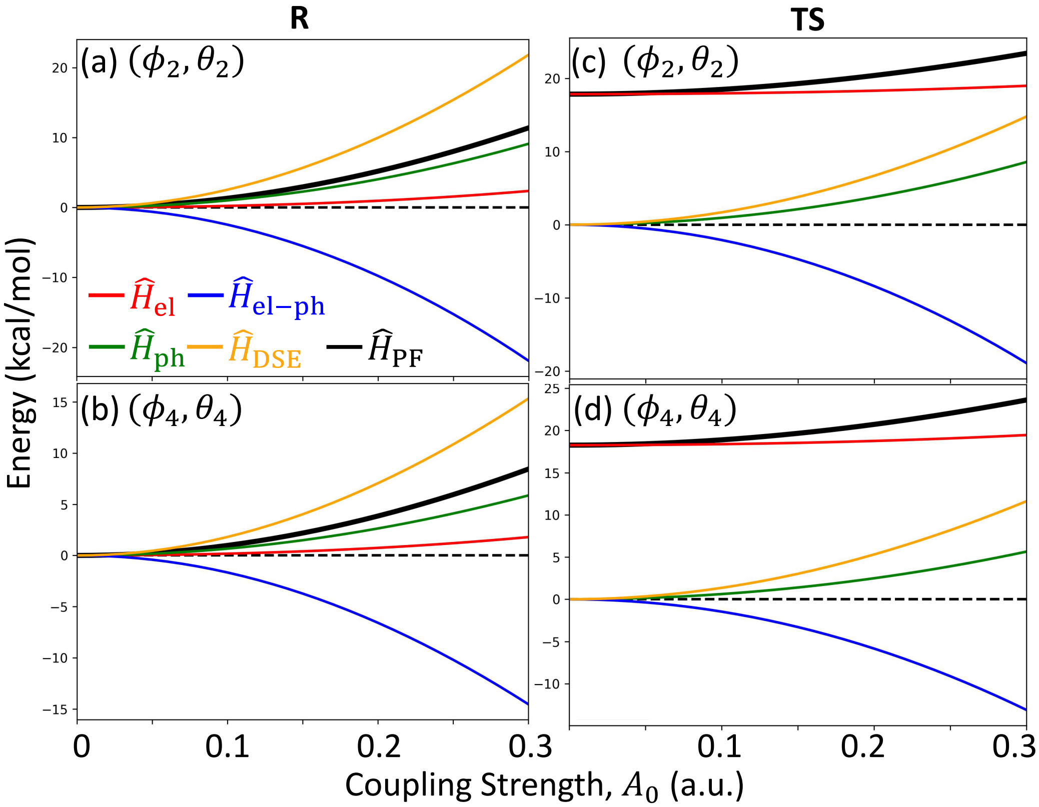

Figure S6: Absolute barrier energy contribution for R and TS to the ground state energy barrier height at minimal difference of $E^{\ddagger} - \mathcal{E}^{\ddagger}$. (a) and (b) show aboslute energy for R; (c) and (d) show aboslute energy for TS. It shows different energy contirbutions towards the total Hamiltonian $\hat{H}_{\mathrm{PF}}$, which is in black solid curved line. Components are $\hat{H}_{\mathrm{el}}$ in red, $\hat{H}_{\mathrm{el-ph}}$ in blue, $\hat{H}_{\mathrm{ph}}$ in green and $\hat{H}_{\mathrm{DSE}}$ in yellow. The horizontal dashed line indicates the uncoupled barrier height (*i.e.*, $A_0 = 0.0$ a.u.). Note that $\hat{H}_{\mathrm{el}}$ starts at the same barrier height as $\hat{H}_{\mathrm{PF}}$ since we treated the molecular Hamitonian at that zero-point level. Overall, $\hat{H}_{\mathrm{DSE}}$ contributes the most to the total energy. The cavity frequency is $\omega_{\mathrm{c}} = 1.5$ eV.
